# 

**Published:** 1895-05

**Authors:** 


### TABLE OF CONTENTS.

ORIGINAL COMMUNICATIONS. PAGE

Diseases of the Maxillary Sinus. By HARRISON ALLEN, M.D., Philadelphia . . . 261

Some Disturbances caused by Diseases of the Teeth. By EDGAR F. STEVENS, D.M.D., Bedford, Mass. . . . 266

Specialism. By REV. NATHAN E. WOOD, D.D., Boston . . . 270

Dental Furnaces. By DR. W. M. SHARP, Binghampton, N. Y. . . . 279

Carving of Block-Teeth. By F. A. CONEY, D.D.S., Doylestown, Pa. . . . 284

On Contour. By Dr. T. G. HUNTINGTON, New York . . . 288

REPORTS OF SOCIETY MEETINGS.

American Society of Dental Science . . . 292

Odontological Society of Pennsylvania . . . 302

Academy of Stomatology . . . 306

EDITORIAL.

The Wrong Use of Words . . . 313

BIBLIOGRAPHY . . . 315

DOMESTIC CORRESPONDENCE.

Reply to Review of Bödecker's Book . . . 319

CURRENT NEWS.

Annual Meeting of the Harvard Odontological Society . . . 320

Dental Society of the State of New York . . . 322

National Association of Dental Examiners . . . 322

Illinois State Dental Society . . . 322

SELECTIONS.

The Value of Peroxide of Hydrogen Preparations . . . 323

